# Acetoclastic versus hydrogenotrophic methanogenesis: defining how pH and alkalinity shape acetate metabolism in a haloalkaliphilic methanogenic community for biomethane production

**DOI:** 10.1007/s00253-026-13725-0

**Published:** 2026-02-16

**Authors:** Beatriz C. Diniz, Ben Abbas, Dimitry Y. Sorokin, Mark C. M. van Loosdrecht, Philipp Zantout-Wilfert

**Affiliations:** 1 Department of Biotechnology, Faculty of Applied Sciences, Delft University of Technology, Van Der Maasweg 9, Delft, 2629 HZ The Netherlands; 2 Urban Water Management, University of Applied Sciences Lübeck, Moenkhofer Weg 239, 23562 Lübeck, Germany; 3 Federal Research Centre of Biotechnology, Winogradsky Institute of Microbiology, Russian Academy of Sciences, Moscow, Russia

**Keywords:** Hydrogenotrophic methanogenesis, Acetoclastic methanogenesis, Haloalkaliphilic microbial community, Alkalinity, Carbon capture

## Abstract

In methanogenic communities, two main pathways drive methanogenesis: acetoclastic methanogenesis, which converts acetate into CH_4_ and CO_2_, and hydrogenotrophic methanogenesis, which reduces CO_2_ with H_2_ to CH_4_. Under high-pH conditions, a shift in dominance from acetoclastic to hydrogenotrophic methanogenesis is often observed. The goal of this work was to identify the pH tipping point for this metabolic shift and to elucidate the influence of alkalinity on this transition in a haloalkaliphilic methanogenic community enriched from anaerobic soda lake sediments. To this end, a haloalkaliphilic microbial community was cultivated across a pH range (8.20–10.00) at three different alkalinities (0.1, 0.6, 1.2 eq/L). Specific qPCR probes were developed to quantify the two dominant methanogens for each catabolism: “*Ca. Methanocrinis natronophilus*” (acetoclastic) and *Methanocalculus alkaliphilus* (hydrogenotrophic). Results showed that the relative abundance of *Methanocalculus* increased with the rise of pH for all alkalinities, with alkalinity exerting a stronger influence than pH. At low alkalinity (0.1 eq/L), *Methanocalculus* abundance doubled from 5.14 ± 1.95% to 9.15 ± 0.77% (pH 8.40–10.35). At moderate alkalinity (0.6 eq/L), it increased from 8.33 ± 1.34% to 47.92 ± 3.76% (pH 8.41–10.00), and at the highest alkalinity (1.2 eq/L), it increased from 6.78 ± 1.06% to 60.25 ± 2.00% (pH 8.26–9.68). 16S rRNA gene amplicon sequencing further identified “*Candidatus* Contubernalis” as a putative syntrophic acetate-oxidizing bacterium likely partnering with *Methanocalculus* in indirect hydrogenotrophic methanogenesis. This work highlights that haloalkaliphilic hydrogenotrophic methanogens offer a promising strategy to integrate CO_2_ capture in alkaline solutions with biomethanation.

**Supplementary Information:**

The online version contains supplementary material available at 10.1007/s00253-026-13725-0.

## Introduction

Atmospheric CO_2_ levels have increased from 280 ppm in the pre-industrial era to 423 ppm in 2024 (Lan et al. 2023). The increase is primarily attributed to anthropogenic emissions, which are linked to climate change (Bajón Fernández et al. 2017), thereby emphasizing the need for effective carbon capture and conversion strategies. One promising solution is the use of hydrogenotrophic methanogens, which reduce carbon dioxide (CO_2_) into methane (CH_4_) using hydrogen (H_2_) as an electron donor (Kleerebezem 2014). In doing so, hydrogenotrophic methanogens allow for the valorization of CO_2_ by transforming it into biomethane.

The use of hydrogenotrophic methanogens for biomethanation, in which dissolved inorganic carbon (DIC) is converted to CH_4_, has been reported throughout the literature. These approaches range from their integration into carbon capture and utilization systems as a desorption approach (Sieborg et al. 2024; Xu et al. 2024) to the addition of H_2_ in conventional anaerobic digestion systems. The latter strategy promotes the dominance of hydrogenotrophic methanogens over acetoclastic methanogens, enabling the efficient conversion of inorganic carbon into methane (Lee et al. 2012).

Acetoclastic methanogens are euryarchaea that catabolize acetate to produce CH_4_ and CO_2_ and are typically dominant in anaerobic digestion systems operating at freshwater, mesophilic, and neutral pH conditions (Conrad 2020). Hydrogenotrophic methanogens, although usually not dominant in such systems, play a crucial role in maintaining process stability under conditions which are not optimal for acetoclastic methanogens to perform acetate metabolism (Mara & Horan 2003). Hydrogenotrophic methanogens can, for example, tolerate up to 11 times higher free ammonia concentrations (NH_3_) than acetoclastic methanogens (Wang et al. 2022). At elevated ammonium conditions, a syntrophic relationship may develop between reversed acetogenic anaerobic acetate-oxidizing bacteria and hydrogenotrophic methanogens, known as syntrophic acetate oxidation (SAO). In this case, bacteria oxidize acetate into H_2_, and hydrogenotrophic methanogens subsequently use the H_2_, together with CO_2_, to produce CH_4_ (Westerholm et al. 2019). Syntrophic acetate-oxidizing bacteria can grow as lithotrophs or heterotrophs. When in a syntrophic consortium with hydrogenotrophic methanogens, they typically oxidize acetate via the reverse Wood–Ljungdahl pathway (Müller et al. 2013), while the use of an oxidative TCA cycle has also been hypothesized (Müller et al. 2015). The reversal of the Wood–Ljungdahl pathway becomes thermodynamically favourable upon removal of produced H_2_ by hydrogenotrophic methanogens.

CO_2_ is not only a gas but is also part of an acid–base system, and at an elevated pH, CO_2_ is speciated towards its soluble ionic forms. In an aqueous solution, ${CO}_{2}\left(g\right)$ is in equilibrium with three different inorganic forms: aqueous carbon dioxide (${CO}_{2}\left(aq\right)$), hydrogencarbonate/bicarbonate (${HCO}_{3}^{-}$), and carbonate (${CO}_{3}^{2-}$). This speciation is often exploited in carbon capture processes, such as in alkaline scrubbing or in the use of bicarbonate absorbents (Xu et al. 2024). The sum of these species is known as dissolved inorganic carbon (DIC) (Eq. 1), and their distribution is pH-dependent, with respective pKa values of 6.33 and 10.33 at 25 °C (Stumm & Morgan 1996). Another important parameter that describes this system is carbonate alkalinity (Eq. 2). In general, alkalinity can be defined as the excess of proton acceptors (bases) over proton donors (acids), and conversely, it is defined as the buffering capacity of a system (Middelburg et al. 2020). For a CO_2_-H_2_O system, the total alkalinity equals to carbonate alkalinity (2); thus, only the carbonic species contribute to the buffering capacity. In this simple system, DIC describes the carbon balance, whilst carbonate alkalinity describes the charge balance.

1 $$DIC= \sum {CO}_{2}=\left[{CO}_{2}\left(aq\right)\right]+\left[{HCO}_{3}^{-}\right]+[{CO}_{3}^{2-}]$$

2 $$carbonate\;alkalinity=\left[{HCO}_3^-\right]+2\left[{CO}_3^{2-}\right]+\left[{OH}^-\right]-\lbrack H^+\rbrack$$

Under alkaline conditions, carbon capture and utilization can be advantageously integrated using alkaliphilic hydrogenotrophic methanogens. Notably, Callander et al. (2024) successfully used a pure culture of an alkalitolerant hydrogenotrophic methanogen (*Methanococcus vannielii*) for this purpose. This organism was cultivated at different pH values (7, 8, and 9) and under varying initial (carbonate) alkalinities between 0 and 0.1 eq/L, corresponding to initial DIC concentrations ranging from 0 to 100 mM. In their study, the highest methane production rate was observed at pH 8 and an alkalinity of 0.06 eq/L. Additionally, it was demonstrated that methanogenesis depended on the total DIC concentration rather than the partial pressure of CO_2_ (pCO_2_). Their work only explored relatively low DIC concentrations, limiting the integration between carbon capture (dissolved CO_2_) and carbon utilization (hydrogenotrophic methanogenesis) to this low range.

The use of a microbial mixed community, rather than a pure culture, would offer greater robustness and broader applicability for the integration of inorganic carbon capture and utilization under alkaline conditions, particularly in complex systems such as anaerobic digesters. An additional advantage of using a mixed community under alkaline conditions is the reported inhibition of acetoclastic methanogenesis, leading to the dominance of the hydrogenotrophic pathway. This metabolic shift is often observed at pH values above 9 (Wormald et al. 2020; Sorokin et al. 2016, 2015; Zhilina & Zavarzin 1994).

Therefore, a haloalkaliphilic mixed microbial community, characterized by its ability to withstand high pH and alkalinity, was used in this work. This haloalkaliphilic community originated from a haloalkaline sequencing-batch reactor treating a complex alkaline organic substrate (pH 8.7 and 0.6 eq/L alkalinity), initially inoculated with soda lake sediments (Diniz et al. 2025). Soda lakes are naturally occurring environments characterized by high and stable pH values (9–11), elevated salinity (> 15–300 g/L), and significant sodium carbonate alkalinity (> 0.2–4 eq/L) (Schagerl & Renaut 2016; Sorokin et al. 2015).

Accordingly, the present study aimed to identify the pH and alkalinity tipping points from acetoclastic to hydrogenotrophic methanogenesis in a methanogenic community and to evaluate the optimal operational window for the conversion of dissolved inorganic carbon (DIC) to biomethane. To this end, a haloalkaliphilic microbial community was tested at various pH values (8.20, 8.5, 9.0, 9.2, 9.5, and 10.0) and initial (Na^+^) carbonate alkalinities (0.1 eq/L, 0.6 eq/L, and 1.2 eq/L), using acetate as a sole substrate.

Triplicate batch tests were prepared for each pH-alkalinity combination and operated until complete acetate depletion. 16S rRNA gene amplicon profiling was employed to assess overall microbial community composition. In addition, to monitor the relative abundance of acetoclastic and hydrogenotrophic methanogens, qPCR probes were developed for the two main methanogens for each respective catabolism: “*Ca.* Methanocrinis natronophilus” (acetoclastic) and *Methanocalculus alkaliphilus* (hydrogenotrophic). Finally, due to methanogenesis operating close to the redox equilibrium, calculations were performed for hydrogenotrophic, acetoclastic, and acetate-oxidizing catabolic pathways across a pH range and tested alkalinities to evaluate their thermodynamic feasibility.

## Materials and methods

### Media, substrate, and inoculum

A range of buffered media to achieve specific initial pH values (8.20, 8.5, 9.0, 9.2, 9.5, 10.0, and 10.5*) and alkalinity levels (0.1, 0.6, and 1.2 eq/L) was employed. For the lowest alkalinity condition (0.1 eq/L), the initial pH was adjusted to 10.5 instead of 10.0 due to concerns about the buffering capacity; this difference is represented by 10.5* (Table 1). All media were composed of NaHCO_3_ and Na_2_CO_3_ in different proportions and concentrations to reach the desired pH alkalinity. The proportions were calculated according to equilibrium constants for HCO_3_^−^ and CO_3_^−2^ (Stumm & Morgan 1996). For the lower pH media (8, 8.5), NaH_2_PO_4_·2H_2_O and Na_2_HPO_4_ were also used and represented 16% of the total Na+ concentration. The different compositions can be found in Table 1. To all buffered media, 5 mM NH_4_Cl, 0.2 mM Na_2_S·9H_2_O, 51 mM of NaCl, and 0.1 mL/L of an alkaline selenium/tungsten solution (NaSeO_3_ 0.15 mM; Na_2_WO_4_ 0.11 mM) were added. Finally, 1 mL/L of the acid trace metal solution described in Pfennig and Lippert (1966) was added. This acid trace metal solution was modified by removing H_3_BO_3_ and substituting MnCL_2_ for MnSO_4_. The carbon substrate used in this work was sodium acetate (NaC_2_H_4_O_2_·3H_2_O) at 15 mM.

**Table 1:** Composition of each medium used for the alkalinity-pH combinations

Expected alkalinity (eq/L)	Initial pH	Buffered media (M)							Substrate (M)	Final Na+ (M)
		NaHCO_3_	Na_2_CO_3_	NaH_2_PO_4_·2H_2_O	Na_2_HPO_4_	NaCl	NH_4_Cl	Na_2_S·9H_2_O	NaC_2_H_4_O·3H_2_O	
0.1	8.20	0.019	0.000	2.98E−04	7.85E−03	0.05	0.005	2E−04	0.015	0.10
	8.5	0.018	0.001	9.56E−05	7.95E−03	0.05	0.005	2E−04	0.015	0.10
	9	0.029	0.003	0	0	0.05	0.005	2E−04	0.015	0.10
	9.2	0.027	0.004	0	0	0.05	0.005	2E−04	0.015	0.10
	9.5	0.021	0.007	0	0	0.05	0.005	2E−04	0.015	0.10
	10.5*	0.002	0.017	0	0	0.05	0.005	2E−04	0.015	0.10
0.6	8.20	0.429	0.004	0.002	0.047	0.05	0.005	2E−04	0.015	0.60
	8.5	0.412	0.013	0.001	0.048	0.05	0.005	2E−04	0.015	0.60
	9	0.445	0.044	0	0	0.05	0.005	2E−04	0.015	0.60
	9.2	0.405	0.064	0	0	0.05	0.005	2E−04	0.015	0.60
	9.5	0.327	0.103	0	0	0.05	0.005	2E−04	0.015	0.60
	10	0.178	0.178	0	0	0.05	0.005	2E−04	0.015	0.60
1.2	8.20	0.924	0.009	0.004	0.094	0.05	0.005	2E−04	0.015	1.20
	8.5	0.887	0.027	0.001	0.095	0.05	0.005	2E−04	0.015	1.20
	9	0.945	0.094	0	0	0.05	0.005	2E−04	0.015	1.20
	9.2	0.861	0.136	0	0	0.05	0.005	2E−04	0.015	1.20
	9.5	0.694	0.220	0	0	0.05	0.005	2E−04	0.015	1.20
	10	0.378	0.378	0	0	0.05	0.005	2E−04	0.015	1.20

The inoculum used in this study originated from the anaerobic digestion of an alkaline complex substrate at an average pH of 8.7 and 0.6 eq/L alkalinity (Diniz et al. 2025). The inoculum used for each alkalinity was analysed through quantitative PCR and 16S rRNA gene amplicon sequencing. The 16S rRNA gene amplicon sequencing results for the inoculum can be found in the supplementary information.

### Batch test preparation

To study the range of pH values (8.20, 8.5, 9.0, 9.2, 9.5, 10.0, and 10.5*) and alkalinities (0.1, 0.6, and 1.2 eq/L), batch tests were prepared for each individual combination. For each combination, three experimental batch tests and four control tests were conducted. Of the controls, two were no-inoculum controls and the other two were no-substrate controls. Each batch test was conducted in a 115-mL serum bottle with a 65 mL liquid volume, of which 3.5 mL was inoculum. Additionally, for each separate experiment, the inoculum had the same volatile solid concentration. All bottles were sealed with butyl rubber stoppers and aluminium crimps. These were made anoxic through a gas exchange setup with three alternating cycles of vacuum and argon gas flushing. Finally, the bottles were placed in a 35 °C oven until acetate depletion.

### Analytical methods

The gas composition of each batch test was measured by pooling the gas volumes from the experimental batch tests and controls into a gas bag, respectively. After collection, the gas bag was injected into a Prima-BT mass spectrometer (Thermo Fisher Scientific, USA) to measure CO_2_, CH_4_, and H_2_. In addition, the total alkalinity (TA) was also measured using the standard titration method in duplicate (Dunnivant 2004) and the pH using a C6010 pH electrode (Consort, Belgium). N-NH_4_ and P-PO_4_ were measured by photometric analysis via the Gallery discrete analyser (Thermo Fisher Scientific, USA), and acetate was measured through a Vanquish HPLC (Thermo Fisher Scientific, USA) with an Aminex HPX-87H column (Bio-Rad, USA).

### Quantitative PCR

Quantitative PCR (qPCR) was performed to quantify the abundance of the main methanogenic genera. These were *Methanocalculus alkaliphilus* and “*Ca.* Methanocrinis natronophilus”, an acetoclastic and a hydrogenotrophic methanogen, respectively. The quantification was done using SYBR Green chemistry on a qTOWER^3^ auto series (Analytik Jena, Germany). The primer sets used in this study are described in Table 2. Both primer sets were developed and calibrated according to the 16S ribosomal RNA gene sequences of *Methanocalculus alkaliphilus* (NCBI, accession number HM053969) and “*Ca.* Methanocrinis natronophilus” (NCBI, accession number KP205578). Additionally, the initial primer development was guided by the work of Yu et al. 2005. For calibration, sequential dilutions were prepared for both sequences, and the known DNA copy numbers were plotted against the corresponding cycle threshold (CT) values. The resulting calibration curves, which were subsequently used in the analysis, are provided in Supplementary Information.

**Table 2:** Characteristics of primer sets used for each respective target groups

Target group	Function	Sequence (5′- > 3′)
*Methanocalculus alkaliphilus*	F primer	ATCGG TACGG GTTGT GGG
	R primer	CACCT AACGC ACATC GTTTA C
*“Ca.* Methanocrinis natronophilus”	F primer	GTAAA CGATG CTCGC TAGGT
	R primer	GGTCT CCACA GTGTA CC

For quantification, the DNA was extracted from each inoculum used and at the end of each batch test (a pooled sample of the triplicate batches was used). The extraction was done with the DNeasy PowerSoil Kit (Qiagen, Germany) following the manufacturer’s protocol. Each 20 µL reaction contained 0.1 µL of the forward and the reverse primer for one of the primer sets (Table 2), 10 µL of iQ SYBR green supermix (Bio-Rad, USA), 8.8 µL PCR-grade water, and 1 µL of the DNA sequence template or extracted DNA sample. The qPCR was performed for 40 cycles with the following thermal profile: 95 °C for 5 s (denaturation), 55 °C for 40 s (annealing), 72 °C for 40 s (extension), and 80 °C for 5 s for signal acquisition. All reactions were run in duplicate dilutions along with no-template controls, and the respective cycle threshold number (Ct) was registered. The raw data for the qPCR results including the initial inoculum results can be found in the supplementary information. Finally, it is important to note that qPCR measurements have limitations, as qPCR amplifies DNA regardless of whether it comes from living, dormant, or dead cells.

### 16S rRNA gene amplicon sequencing analysis

DNA was extracted from each inoculum and from a pooled sample of triplicates collected at the end of each batch test. This extraction is described in the previous section. The total genome DNA was further extracted using the CTAB method, and the DNA concentration and purity were monitored on 1% agarose gels. According to this concentration, DNA was diluted at 1 ng/L. The V4–V3 region of the 16S ribosomal RNA gene was amplified by PCR using the primers 341 F (5′ CCTAYGGGRBGCASCAG 3′) and 806R (5′ GGACTACNNGGGTATCTAAT 3′). The PCR reactions were performed with 15 µL High-fidelity PCR master mix (Phusion, USA), where 2 µM of forward and reverse V3–V4 rRNA primers was added and 10 ng of template DNA. The PCR products were identified by 2% agarose gel and purified with the gel extraction kit (Qiagen, Germany). Sequencing libraries were then generated using the TruSeq DNA PCR-Free preparation kit (Illumina, USA). The library was sequenced on a Novaseq platform (Illumina, USA), and 250 bp paired-end reads were generated. The paired-end reads were then assigned to samples based on their respective barcode and truncated by cutting off the barcode and primer sequence.

Using the DADA2 pipeline (Callahan et al. 2016), paired‐end reads were merged and quality-filtered to obtain high-quality clean tags (Bokulich et al. 2013), with a minimum Phred score of 30. Chimeric sequences were removed, and then, the sequences were clustered into ASVs. After clustering, the SILVA 138.2 database was used to annotate the taxonomy information (Bolyen et al. 2019; Yilmaz et al. 2014). Downstream analyses and plotting were carried out in R using phyloseq (McMurdie & Holmes, 2013). . Raw reads have been deposited in ENA under project accession number PRJEB104023.

### Thermodynamic analysis

The non-ideal Gibbs free energy ($\Delta {G}^{1}$) was calculated for the acetoclastic, hydrogenotrophic, and acetate oxidation catabolism (Table 3) across a pH range between 6 and 10. $\Delta {G}^{1}$ can be calculated with the following formula:

$${\Delta G}_{Reaction}^{1}= {\Delta G}_{Reaction}^{0}+R\bullet T\bullet \sum_{i=1}^{n}{Y}_{Si}^{Reaction}\bullet \mathrm{ln}({\gamma }_{Si}\bullet {c}_{Si})(3)$$

where ${\Delta G}_{Reaction}^{0}$ represents the standard Gibbs free energy for a given reaction (Table 3); R represents the gas constant; T is the temperature at which the reaction occurred; ${Y}_{Si}^{Reaction}$ is the stochiometric value for the reactant Si for a given reaction; ${\gamma }_{Si}$ is the initial activity for reactant Si; and finally, ${c}_{Si}$ is the initial concentration for reactant Si.

**Table 3:** Catabolic reactions for acetoclastic methanogenesis, hydrogenotrophic methanogenesis and acetogenic acetate oxidation

Catabolism	Reaction	${\Delta G}_{Reaction}^{0}$ (Kj/mol)
Acetoclastic methanogenesis	$-1{C}_{2}{H}_{3}{O}_{2}^{-}-1{H}^{+}+1{CO}_{2}+1{CH}_{4}$	−65.83
Hydrogenotrophic methanogenesis	$-{4H}_{2}-{1CO}_{2}+1{CH}_{4}+2{H}_{2}O$	−140.82
Acetate oxidation	$-1{C}_{2}{H}_{3}{O}_{2}^{-}-{2H}_{2}O-1{H}^{+}+4{H}_{2}+2{CO}_{2}$	75

For the purpose of these calculations, several assumptions were made. Firstly, only dissolved CO_2_ was considered among the inorganic carbon species. Its initial concentration was calculated according to the bicarbonate/carbonate equilibrium at a given pH ($pK{a}_{{HCO}_{3}^{-}}=$ 6.33 and $pK{a}_{{CO}_{3}^{2-}}=$ 10.33 at 25 °C). The initial concentration of acetate was fixed for all calculations at 15 mM. Additionally, it was assumed that anabolism (growth) was negligible, and all acetate was used for the catabolism. The initial hydrogen and methane concentrations were assumed to correspond to the value measured in abiotic controls with the liquid-phase concentration determined using Henry’s law (${H}_{{H}_{2}}=$ 0.78 mmol/kg*bar, ${H}_{{CH}_{4}}=$ 1.4 mmol/kg*bar): H_2_: 0.00285 mM and CH_4_: 0.015 mM. All activity coefficients were assumed to be 1, and Gibbs free energy values were adjusted for the experimental temperature of 35 °C. The script for all calculations can be found in supplementary information.

## Results

### General batch operation

An acclimatized haloalkaliphilic anaerobic community was cultured in triplicate batch tests across a pH range (8.2–10.0) and at three alkalinity levels (0.1, 0.6, 1.2 eq/L). It is important to note that the final Na^+^ concentration equals the alkalinity (e.g. 0.1 eq/L = 0.1 mol/L Na^+^). Each batch was supplied with 15.7 ± 1.2 mM of acetate and operated until acetate was fully depleted.

For all batch tests, acetate uptake occurred linearly, and for each alkalinity, the acetate consumption rate decreased on average 30% for higher pH values (Table 4). Additionally, for the batch tests at 0.1 eq/L alkalinity, the acetate consumption rates were approximately three times higher than the rates observed for 0.6 eq/L and 1.2 eq/L alkalinities.

In Table 4, three pH values are reported for each batch test: buffer pH, initial pH, and final pH. All values were measured in triplicate, with deviations below ± 0.03. The buffer pH represents the pH of the prepared buffer for the expected pH-alkalinity combination studied, before the addition of acetate, ammonia, and the inoculum. The initial pH represents the average pH of the batch tests at the start point, while the final pH represents the average pH at the endpoint. As expected, for the lowest alkalinity studied (0.1 eq/L), the largest deviation between initial and final pH was observed, with an average change of 0.3 pH units. For alkalinity 0.6 eq/L, the average deviation was 0.06 pH units, and for 1.2 eq/L alkalinity, it was 0.03 pH units. For most conditions, this deviation corresponded to a decrease in pH, attributed to microbial CO_2_ production. However, for the 0.1 eq/L alkalinity at an initial pH of 8.40, the final pH was higher: 8.72, which is hypothesised to be a result of the lower buffering capacity combined with the initial pH of the inoculum—8.7. The final gas composition for each pH-alkalinity combination is reported in Table 4. Here, it is possible to see that the CO_2_ partial pressure decreased with the increase in pH, due to the shift in speciation towards soluble carbonic species.

**Table 4:** Measured parameters in the batch tests under each pH-alkalinity condition: pH values, gas composition (%), and acetate consumption rate (mmol/L/day)

Final average alkalinity (eq/L)	pH^a^			Final gas composition (%)			Incubation time (day)	Acetate consumption rate (mmol/L/day)^b^
	Buffer	Initial	Final	CO_2_	CH_4_	H_2_		
0.09 ± 0.01	8.22	8.40	8.72	3.93 ± 0.54	95.91 ± 6.59	0.18 ± 0.01	23	0.71 ± 0.02
	8.39	9.01	8.92	4.19 ± 1.42	95.63 ± 5.04	0.18 ± 0.01	23	
	9.16	9.15	9.01	2.89 ± 0.41	96.96 ± 1.95	0.16 ± 0.02	23	
	9.44	9.40	9.13	2.21 ± 0.91	97.62 ± 3.38	0.16 ± 0.09	23	
	9.93	9.87	9.43	1.87 ± 0.77	98.97 ± 7.75	0.17 ± 0.02	30	0.54 ± 0.01
	10.48	10.35	9.77	1.49 ± 0.70	98.26 ± 0.24	0.25 ± 0.03	30	
0.58 ± 0.03	8.25	8.41	8.41	50.28 ± 0.27	49.63 ± 3.57	0.09 ± 0.05	76	0.23 ± 0.02
	8.61	8.64	8.59	29.85 ± 0.13	70.03 ± 7.07	0.12 ± 0.07	64	
	9.04	8.99	8.94	18.45 ± 1.13	81.41 ± 7.49	0.14 ± 0.06	64	
	9.24	9.17	9.13	9.96 ± 0.45	89.89 ± 5.80	0.15 ± 0.06	64	
	9.52	9.45	9.38	5.58 ± 0.27	94.26 ± 2.84	0.16 ± 0.05	64	
	10.07	10.00	9.86	1.45 ± 0.91	98.38 ± 3.96	0.17 ± 0.05	103	0.15
1.34 ± 0.10	8.04	8.26	8.24	49.30 ± 3.40	50.62 ± 3.21	0.08 ± 0.05	68	0.23 ± 0.01
	8.27	8.40	8.40	-	-	-^c^	68	
	8.70	8.63	8.60	34.25 ± 3.00	65.64 ± 3.06	0.11 ± 0.04	75	
	8.90	8.81	8.80	20.58 ± 0.90	79.28 ± 0.27	0.13 ± 0.02	75	
	9.20	9.10	9.08	13.96 ± 0.51	85.89 ± 0.92	0.15 ± 0.03	92	0.16 ± 0.02
	9.70	9.68	9.62	2.06 ± 0.31	97.76 ± 0.17	0.17 ± 0.01	114	

^a^pH values were measured in triplicate; all deviations were below ± 0.03. Buffer represents the pH value of buffer without the addition of acetate, ammonia, and inoculum.

^b^Acetate consumption rate changed for higher pH values.

^c^Due to technical issues, it was not possible to measure the gas composition for this datapoint.

### Dynamics of the microbial community

To monitor the relative abundance of acetoclastic and hydrogenotrophic methanogens at the end of each batch test, quantitative PCR (qPCR) probes were developed targeting the dominant methanogens associated with each respective catabolic pathway in the inoculum (acetoclastic and hydrogenotrophic methanogenesis). For the acetoclastic methanogenesis pathway, this was the “*Ca.* Methanocrinis natronophilus” (family *Methanotrichaceae*), and for the hydrogenotrophic methanogenesis pathway, the dominant methanogen targeted was *Methanocalculus alkaliphilus* (family *Methanocalculaceae*). The inoculum used in this work originated from a laboratory-scale anaerobic digester fed with a complex organic substrate (pH 8.7 and 0.6 eq/L alkalinity). This reactor was originally inoculated with soda lake sediments (Diniz et al. 2025).

**Fig. 1:**
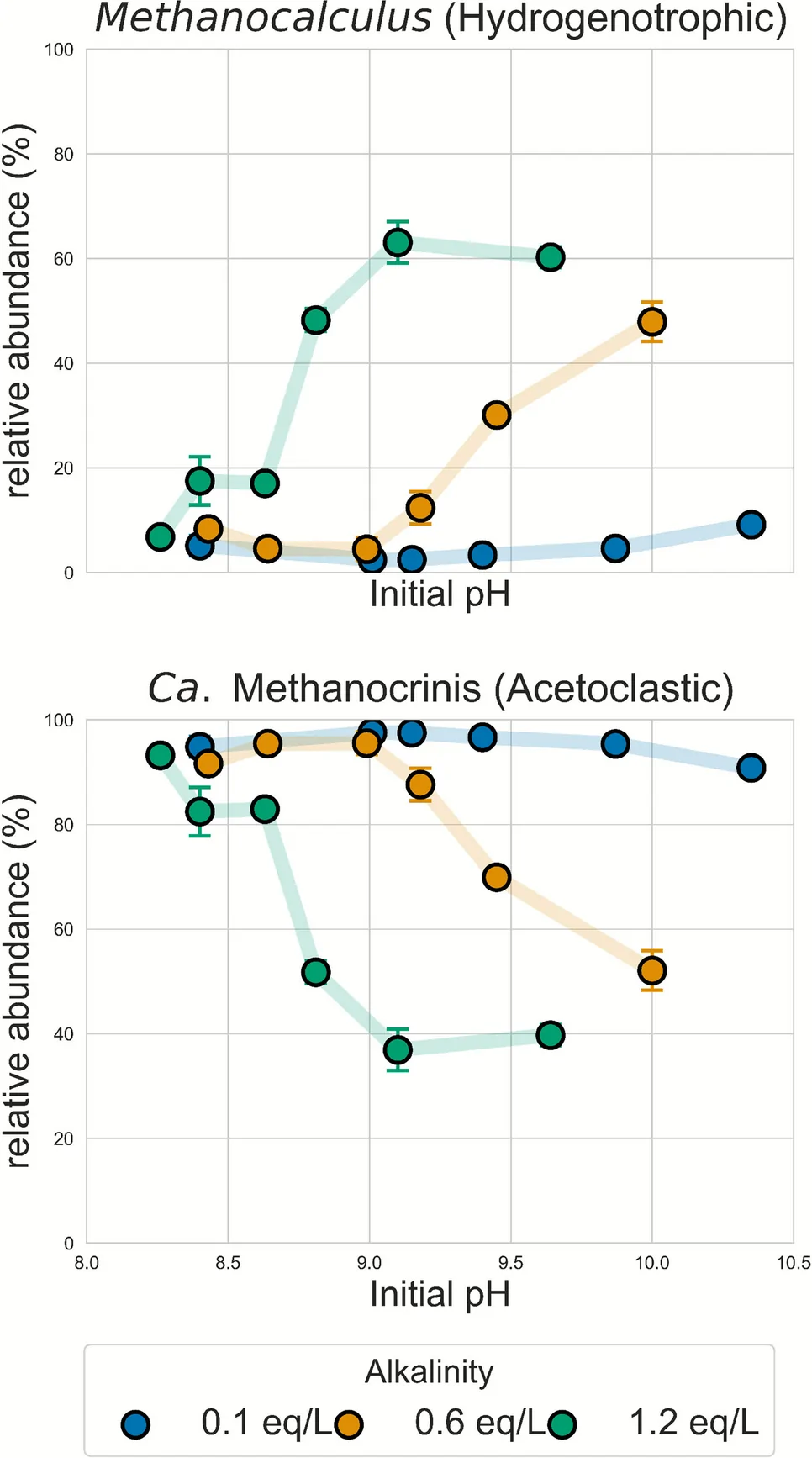
Relative abundance (%) of *Methanocalculus alkaliphilus* (top) and “*Ca.* Methanocrinis natronophilus” (bottom) across the studied pH range and alkalinity levels (0.1 blue circle, 0.6 orange circle, and 1.2 green circle eq/L), as determined by quantitative PCR (qPCR)

Figure 1 shows the relative abundance (%) of *Methanocalculus alkaliphilus* and “*Ca.* Methanocrinis natronophilus” across the studied pH range and alkalinities. The results indicate that the relative abundance of *Methanocalculus* increased overall with higher initial pH values, and that this increase began at a lower initial pH when the alkalinity was higher. For instance, at 0.1 eq/L alkalinity, the relative abundance of *Methanocalculus* increased 1.8-fold from lowest pH to the highest. At 0.6 eq/L alkalinity, it increased 5.7-fold, and at 1.2 eq/L alkalinity, it increased 9.3-fold from pH 8.26 to pH 9.10 and stabilized at pH 9.68.

**Fig. 2:**
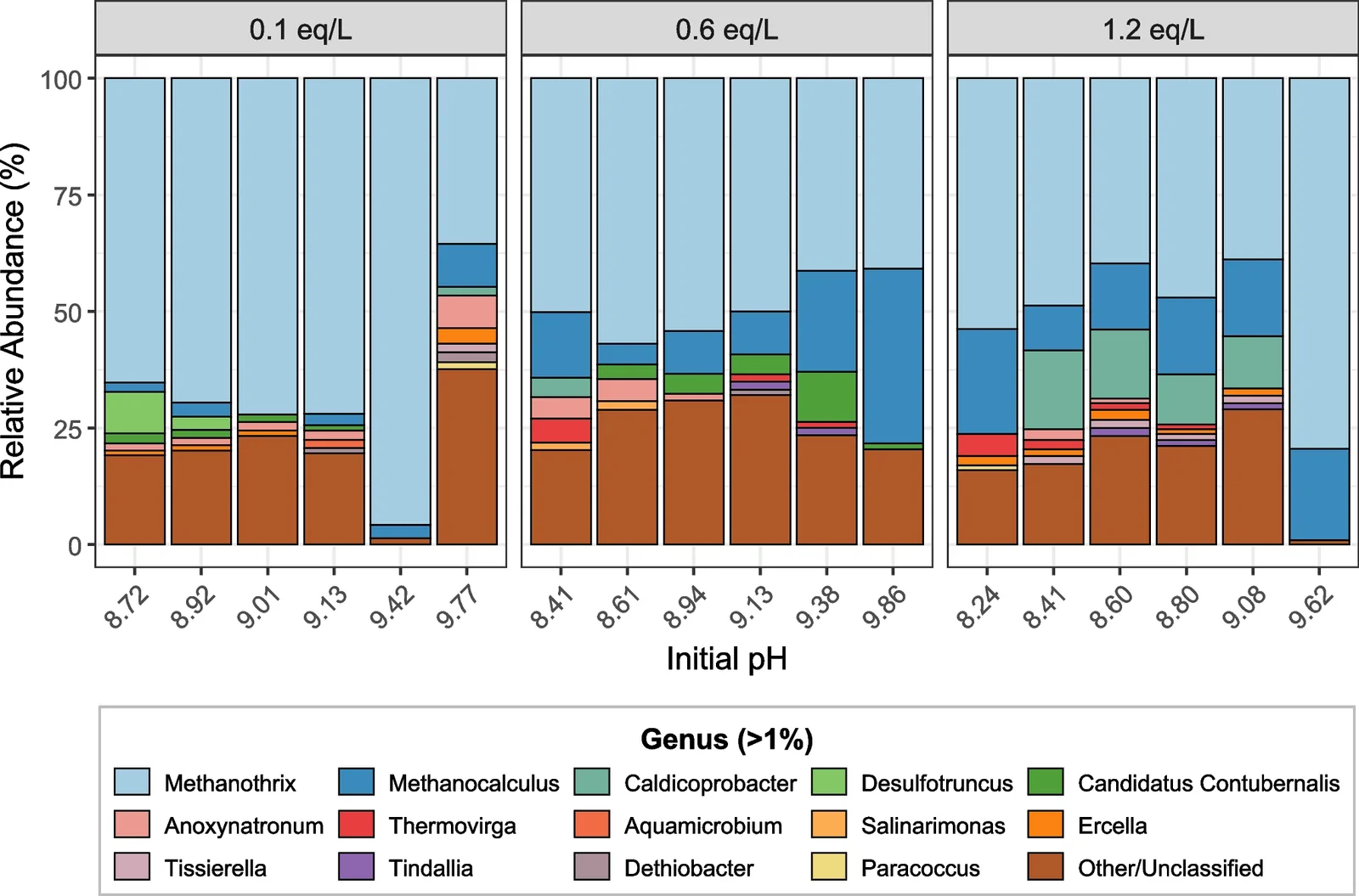
Relative abundance of bacterial and archaeal genera for each pH-alkalinity combination. Panels correspond to alkalinity levels: 0.1 eq/L (left), 0.6 eq/L (middle), and 1.2 eq/L (right), with samples in each panel ordered from lowest to highest initial pH. Results are shown at genus level; genera with < 1% abundance and unclassified taxa are grouped as “other/unclassified”. It is important to note that the *Methanothrix* genus includes “*Ca.* Methanocrinis natronophilus”

Following the qPCR results, which showed clear shifts in the abundance of key methanogens, the microbial community was further analysed using 16S rRNA gene amplicon sequencing, with a particular focus on detecting potential syntrophic acetate-oxidizing bacterium. Each batch test was sampled at the end of its run, and the dominant genera were identified in each sample (Fig. 2).

In Fig. 2, the two dominant genera across all samples were *Methanothrix* and *Methanocalculus*. It is important to note that the alkaliphilic branch of *Methanothrix* was recently reclassified into “*Ca.* Methanocrinis natronophilus” (Khomyakova et al. 2023). Hence, in this case, the *Methanothrix* genus observed in Fig. 2 corresponds to the “*Ca.* Methanocrinis” genus. This was further confirmed by an NCBI BLAST analysis, which showed a 100% match between the amplicon sequence variants (ASVs) assigned to *Methanothrix* and “*Ca.* Methanocrinis natronophilus”. Therefore, the two main genera observed in all samples were the same taxa targeted in the qPCR analysis (Fig. 1).

Figure 2 shows that the relative abundance of *Methanocalculus* increased with higher initial pH values at 0.1 and 0.6 eq/L alkalinity, consistent with the qPCR results. In contrast, at 1.2 eq/L alkalinity, no clear trend with pH was observed. The main bacterial genera observed were *Caldicoprobacter*, *Desulfotruncus*, and “*Ca.* Contubernalis” (Fig. 2). The genus *Caldicoprobacter*, which belongs to the order *Clostridiales*, comprises thermophilic, anaerobic, and xylanolytic bacteria (Bouanane-Darenfed et al. 2014; Bouznada et al. 2024), and was predominantly observed at 1.2 eq/L alkalinity (Fig. 2). *Desulfotruncus* is a known sulphate-reducing bacterium often found in anoxic marine sediments (Watanabe et al. 2020). In this work, it was only present at the lowest alkalinity (0.1 eq/L) and pH (8.72, 8.92). Finally, “*Candidatus* Contubernalis” includes known alkaliphilic syntrophic acetate-oxidizing bacteria found in moderately saline soda lakes (Sorokin et al. 2016; Zhilina et al. 2005). “*Ca.* Contubernalis” was observed throughout 0.1 eq/L and 0.6 eq/L alkalinity

### Thermodynamic analysis

**Fig. 3:**
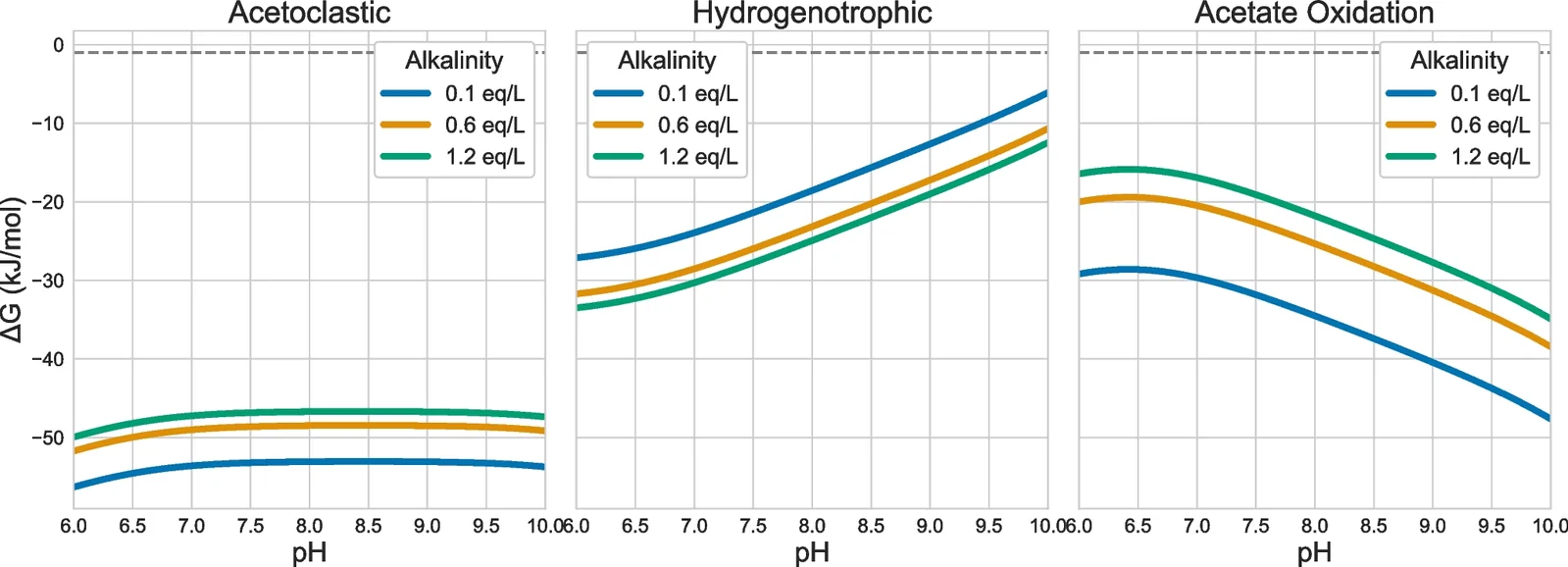
Gibbs free energy (Δ*G*—Kj/mol) for the acetoclastic (left), hydrogenotrophic (middle), and acetate oxidation (right) catabolic reactions. Δ*G* was calculated across a pH range (6–10) and for the three studied alkalinities (0.1eq/L: blueline, 0.6eq/L:orangeline, and 1.2 eq/L: green line)

To determine whether the metabolic shifts observed in the microbial community were thermodynamically constrained, the Gibbs free energy (Δ*G*) was calculated across the experimental pH range and at the tested alkalinity levels (0.1 eq/L, 0.6 eq/L, and 1.2 eq/L). The calculations were done for the main catabolic pathways observed in the microbial community, hydrogenotrophic methanogenesis, acetoclastic methanogenesis, and acetate oxidation (Table 3), and were adjusted for the initial acetate, hydrogen, and methane concentration. A detailed description of the calculations used, as well as the underlying assumptions and parameters, is provided in materials and methods, and the final results are provided in Fig. 3.

Figure 3 shows that across all calculated scenarios, acetoclastic methanogenesis is thermodynamically more favourable compared to the hydrogenotrophic methanogenesis and syntrophic acetate oxidation. Within the acetoclastic catabolism, the changes in pH did not significantly affect the Gibbs free energy (Δ*G*), whereas the increase in alkalinity levels led to higher Δ*G* values, making it more unfavourable for higher alkalinities. For hydrogenotrophic methanogenesis at a fixed pH value, the increase in alkalinity levels led to lower Δ*G* values, making this catabolism more thermodynamically favourable at higher alkalinity levels. In parallel, for this catabolism, the increase in pH led to the increase in Δ*G* values, making hydrogenotrophic methanogenesis thermodynamically more unfavourable at higher pH values. Finally, for acetate oxidation, the increase in pH led to the decrease in Δ*G* values, and the increase in alkalinity led to its increase. Therefore, acetate oxidation becomes more thermodynamically favourable with the increase in pH and decrease in alkalinity.

## Discussion

### General batch operation

The current work demonstrated full acetate depletion and CH_4_ production under all studied conditions (pH, 8.2–10; alkalinity, 0.1 eq/L, 0.6 eq/L, and 1.2 eq/L), confirming the activity and broad operational range of the haloalkaliphilic microbial community used. For each alkalinity, two distinct acetate consumption rates were calculated according to the operational pH (Table 4). Overall, the acetate consumption rates measured in this study were consistent with the range previously reported for the same inoculum: 0.25–0.60 mmol/L/day (Diniz et al. 2025). On average, the consumption rate (mmol/L/day) slowed down by 30% for the final pH values studied for each alkalinity (Table 4). This decrease could be a result of the shift observed towards hydrogenotrophic methanogenesis at higher pH values (Fig. 1). It has been shown that syntrophic acetate oxidizers, in association with hydrogenotrophic methanogens, generally exhibit slower growth than acetoclastic methanogens, potentially resulting in lower acetate consumption rates (Demirel & Scherer 2008; Gehring et al. 2016). Additionally, this rate was around three times higher for 0.1 eq/L alkalinity compared to 0.6 and 1.2 eq/L alkalinities. This effect may have been caused by inhibition from the increase in Na+ concentration. An increase in alkalinity resulted in a parallel increase in the overall Na+ concentration, which in turn increased the salinity and the osmotic stress. 

The headspace CO_2_ content (%) was also affected by the changes in pH and alkalinity. Specifically, CO_2_ decreased as the initial pH increased at all tested alkalinity levels. This trend can be attributed to the shift in CO_2_ speciation towards bicarbonate and carbonate at higher pH values, causing inorganic carbon to remain in the liquid phase (Eq. 1). This highlights the intricate balance within the system: lower pH values accelerate acetate consumption rates, leading to more rapid biogas production, whereas higher pH values enhance CH_4_ purity in the produced gas. In future research, this trade-off should be considered when selecting the operating pH, depending on the optimization goal, whether maximizing substrate conversion or achieving higher CH_4_ purity.

Finally, it was also observed that the overall CO_2_ content (%) increased with alkalinity, even as it decreased with pH (Table 4). This can be attributed to the overall increase in dissolved inorganic carbon (DIC), which results in a greater amount of CO_2_ present overall. This data shows that the haloalkaliphilic community used was able to adapt and shift to the different pH alkalinity conditions imposed within the boundaries of the sodium bicarbonate-carbonate buffer system.

### Dynamics of the microbial community

This study aimed to determine the pH and alkalinity tipping points at which a haloalkaliphilic methanogenic community shifted from acetoclastic to hydrogenotrophic methanogens. These tipping points were quantified through qPCR by targeting key methanogens (Fig. 1). Results indicated that the relative abundance of *Methanocalculus alkaliphilus*, a hydrogenotrophic methanogen, increased with the rise in initial pH in comparison to the “*Ca.* Methanocrinis natronophilus”, an acetoclastic methanogen. This pH-driven shift towards hydrogenotrophic methanogens aligns with previous reports (Wormald et al. 2020; Zhilina & Zavarzin 1994). Notably, the increase observed in Fig. 1 was not uniform across all alkalinities, as a higher alkalinity led to a higher relative abundance of the *Methanocalculus* genus, with this increase beginning at a lower pH value.

This shows that alkalinity had a clearly stronger impact on the methanogenic community than pH. This could have been an effect of the parallel increase in salinity (Na+ concentration), but also an effect of the overall increase in dissolved inorganic carbon (DIC) and subsequent CO_2_ availability. Hydrogenotrophic methanogens have been shown to grow and withstand higher salinity levels than acetoclastic methanogens (Oren 1999), which might have led to the outcompeting of the acetoclastic methanogens at higher salinity levels. Additionally, in Table 4, it is possible to see that for the same pH, the pCO_2_ is higher for 0.6 eq/L and 1.2 eq/L alkalinity compared with 0.1 eq/L, leading to more substrate availability for the hydrogenotrophic methanogenesis pathway. In fact, Chen et al. 2019 showed that the growth rate of *Methanosarcina barkeri* performing hydrogenotrophic methanogenesis was influenced by the DIC concentration, where an increase in the DIC concentration from 0.44 to 6.46 mM led to a 47-fold increase in the growth rate. Hence, the increase in abundance of hydrogenotrophic methanogens at higher alkalinities might have been caused by a combined effect of the increase in salinity and DIC.

In Fig. 2, the microbial community was further analysed beyond its methanogens using 16S rRNA gene amplicon sequencing, with the main goal of detecting the syntrophic acetate-oxidizing bacterium involved in this shift from acetoclastic to hydrogenotrophic methanogenesis. The three main bacterial genera observed in Fig. 2 throughout all samples were *Caldicoprobacter*, *Desulfotruncus*, and “*Ca.* Contubernalis”*.* Notably, “*Ca.* Contubernalis alkalaceticum” and related bacteria detected in acetate-dependent methanogenic enrichments were found in moderately saline soda lakes (Sorokin et al. 2016; Zhilina et al. 2005). This genus was detected at high relative abundance under 0.6 eq/L alkalinity, while remaining relatively low at both 0.1 eq/L and 1.2 eq/L (< 1%). Notably, its highest abundance corresponded to a datapoint around the optimal growth conditions reported by Sorokin et al. (2016), at pH 9.7 and Na+ salinity between 0.3 and 1 M. Additionally, the observed *Desulfotruncus* genus is a marine sulphate-reducing bacterium that utilises fatty acids and H_2_. In this work, *Desulfotruncus* was only observed at the lowest alkalinity and lowest pH values (Fig. 2), as this genus is reported to be neutrophilic (Watanabe et al. 2020). The *Caldicoprobacter* genus was observed at high abundance levels under 1.2 eq/L alkalinity. This member of the class *Clostridia* includes metabolically versatile anaerobes with the ability to degrade complex carbohydrates under thermophilic conditions (Bouznada et al. 2024), and it has also been found at high salinity conditions. In fact, an NCBI BLAST analysis revealed that the highest match for the amplicon sequence variants (ASVs) associated with this genus was previously linked to anaerobic granulation under high salinity conditions (accession MN414334). However, the specific role of *Caldicoprobacter* at 1.2 eq/L alkalinity remains to be elucidated.

In Fig. 2, archaeal genera were also observed. The two dominant genera across all samples, *Methanothrix* and *Methanocalculus*, were the same taxa targeted in the qPCR analysis, since the alkaliphilic branch of *Methanothrix* was reclassified to “Ca. Methanocrinis natronophilus” (Khomyakova et al. 2023). Despite observing the same general trends with increasing pH at alkalinities of 0.1 eq/L and 0.6 eq/L, the relative abundance values of *Methanocalculus alkaliphilus* and “Ca. Methanocrinis natronophilus” differed between the qPCR results and the 16S rRNA gene amplicon sequencing results. This discrepancy may be attributed to biases inherent to 16S rRNA amplicon sequencing, including primer-dependent amplification efficiencies and differences in 16S rRNA gene copy numbers among taxa (Poretsky et al. 2014). Notably, with the primers used, coverage differed between archaea (up to 91.48%) and bacteria (up to 89.82%) (Tahon et al. 2021).

Finally, it is important to note that 16S rRNA amplicon sequencing-based abundance estimates do not allow for the quantification of the individual contributions of specific methanogens to CH_4_ production. Future studies should address this limitation by applying approaches such as ^13^C-methyl acetate-based stable isotope probing (SIP), in which labelled acetate can be tracked through the methanogenic community (Groninga et al. 2025).

### Thermodynamic analysis

Acetate-dependent methanogenesis operates close to thermodynamic equilibrium, making Gibbs free energy (Δ*G*) calculations essential for evaluating pathway feasibility (Dolfing et al. 2010). Figure 3 shows that changes in alkalinity and pH influenced the potential Δ*G* associated with methanogenesis. An increase in alkalinity made hydrogenotrophic methanogenesis more exergonic, while simultaneously disfavouring both acetoclastic methanogenesis and acetate oxidation. Conversely, higher pH reduced the thermodynamic feasibility of hydrogenotrophic methanogenesis and enhanced that of acetate oxidation, whereas acetoclastic methanogenesis was largely unaffected. However, these thermodynamic tendencies were not fully reflected in the trends observed in Fig. 1 as acetoclastic methanogenesis remained the most thermodynamically favourable catabolism under all tested conditions. This mismatch between predicted thermodynamics and observed community changes indicates that the transition from acetoclastic to hydrogenotrophic methanogenesis is not purely thermodynamically controlled; instead, kinetic limitations, enzyme-level regulation, or microbial interactions likely co-determine the dominant catabolic route.

Several mechanisms could account for the discrepancy between feasibility and outcome. The maintenance energy is not taken into account in these calculations, and it is likely to increase in extreme environments such as high pH and alkalinity. It has been shown that acetoclastic methanogens are more sensitive to inhibitory conditions in comparison to hydrogenotrophic methanogens, such as at high salinity (Oren 1999) and at high ammonia levels (Wang et al. 2022). With the increase in pH, total ammonia will be speciated towards free ammonia (NH_3_), which has been shown to inhibit methanogenic communities (Moerland et al. 2021).This suggests that hydrogenotrophic methanogens might have additional adaptation strategies to these potentially inhibitory conditions. Together, these factors provide a plausible biochemical reasoning for the observed shift towards hydrogenotrophic methanogenesis, despite Δ*G* trends that favour acetoclastic catabolism.

## Conclusions

This study aimed to identify the pH and alkalinity tipping points for the shift from acetoclastic to hydrogenotrophic methanogenesis in a haloalkaliphilic methanogenic community. The results showed that alkalinity (and salinity), not pH alone, was the primary driver for this shift, and that hydrogenotrophic methanogenesis dominance can be selectively induced by elevating alkalinity and/or pH. Additionally, it was shown that the observed shift is not predicted through thermodynamic calculations, indicating that the biochemical constraints play a role in this dynamic. Haloalkaliphilic hydrogenotrophic methanogens offer a promising and robust strategy to integrate CO_2_ capture in alkaline solutions with biomethanation, and its potential should be further explored.

## Supplementary Information

Below is the link to the electronic supplementary material.

ESM1 Summary (XLSX 12.0 KB)

ESM2 (TXT 183 KB)

ESM3 (XLSX 96.7 KB) 

## Data Availability

Data will be made available on request.
